# Effect of periodontal therapy on COPD outcomes: a systematic review

**DOI:** 10.1186/s12890-021-01429-2

**Published:** 2021-03-18

**Authors:** Ioulianos Apessos, Athanasios Voulgaris, Michalis Agrafiotis, Dimitrios Andreadis, Paschalis Steiropoulos

**Affiliations:** 1 Department of Oral Surgery, Surgical Implantology and Oral Radiology, School of Health Sciences, School of Dentistry, Aristotle University of Thessaloniki, 54124 Thessaloniki, Greece; 2 Department of Dentistry, 424 General Military Training Hospital, Thessaloniki, Greece; 3 Department of Pneumonology, Medical School, Democritus University of Thrace, Alexandroupolis, Greece; 4 MSc Program in Sleep Medicine, Medical School, Democritus University of Thrace, Alexandroupolis, Greece; 5 Department of Respiratory Failure, General Hospital “G. Papanikolaou”, Aristotle University of Thessaloniki, Thessaloniki, Greece; 6 Department of Oral Medicine/Pathology, School of Dentistry, Aristotle University of Thessaloniki, Thessaloniki, Greece

**Keywords:** Chronic obstructive pulmonary disease, COPD exacerbation, Periodontal therapy, Oral health, Systematic review

## Abstract

**Background:**

Latest evidence suggests that periodontitis is prevalent among patients with chronic obstructive pulmonary disease (COPD), while recent studies have also reported a potential benefit of periodontal treatment on several COPD outcomes. This systematic review aims to determine the impact of periodontal treatment on exacerbation rate, lung function and quality of life of COPD patients.

**Methods:**

A systematic search of electronic databases of PubMed, Scopus, Virtual Health Library, ScienceDirect, Wiley Online Library, Web of Science, ProQuest Dissertation and Theses Global and Google Scholar was conducted. Search restricted to studies involving human subjects which were published from January 2000 to March 2020 in English language. Distiller Systematic Review software was used for data management. Risk of bias was assessed using Risk of Bias 2 (RoB2) and Risk of Bias for non-randomized studies of intervention (ROBINS-I) tools. Overall quality of evidence was judged based on Grading of Recommendations Assessment, Development and Evaluation working group methodology.

**Results:**

Out of 1442 articles retrieved, 7 full text articles were included in the review. Limited evidence suggests that periodontal treatment in patients with COPD and periodontitis is associated with reduced exacerbation frequency and a slower lung function decline rate, while its effects on quality of life remain unclear. In addition, periodontal treatment in COPD is associated with lower hospitalization rates and reduced all-cause mortality. Significant methodological differences were noted amongst included studies, while very low-to-moderate overall quality of evidence was demonstrated.

**Conclusions:**

Although it is reasonable to advise COPD patients not to neglect their dental health, further studies are warranted to determine the role of periodontal therapy on COPD clinical outcomes.

**Trial Registration**: PROSPERO 2020 (CRD42020158481). https://www.crd.york.ac.uk/prospero/display_record.php?ID=CRD42020158481

**Supplementary Information:**

The online version contains supplementary material available at 10.1186/s12890-021-01429-2.

## Background

Chronic obstructive pulmonary disease (COPD) is characterized by the presence of partially reversible airflow limitation with associated symptoms and history compatible with exposure to tobacco and other noxious particles or gases [1]. COPD is a common, preventable and treatable disease and constitutes a major cause of morbidity and mortality [1] with an increasing burden placed the 3^rd^ leading cause of death in 2020 [2]. COPD exacerbations are complex events, triggered by respiratory viral or bacterial pathogens, as well as environmental factors such as pollution and ambient temperature, and often require treatment changes or even hospital admission. In addition, they can negatively impact on health status, hospitalization rates and disease progression and ultimately result in poorer outcomes [3, 4].

COPD often coexists with several diseases that may significantly affect patients’ prognosis [5]. Cardiovascular disease (CVD), hypertension, lung cancer, metabolic syndrome, and obstructive sleep apnea are among the most prevalent comorbidities in COPD patients [5, 6]. Periodontitis is a chronic multifactorial inflammatory disease associated with dysbiotic plaque biofilms and is characterized by progressive destruction of the tooth supporting tissue [7]. Periodontitis can result in tooth loss, while it may also affect chewing function and aesthetics, leading ultimately to impaired quality of life. Moreover, periodontitis accounts for a substantial proportion of edentulism and masticatory dysfunction cases, resulting in significant dental care costs [7]. Overall, periodontitis exerts a major burden on public health systems [8].

Periodontitis and COPD are chronic progressive inflammatory diseases that share common risk factors such as smoking, age and lower socioeconomic status [9, 10]. Evolving evidence points towards an association between COPD, oral health and periodontal disease [8]. In a meta-analysis of 14 observational studies, the presence of periodontitis had an odds ratio (OR) of 2.08 for developing COPD [11]. On the other hand, when compared to the general population, COPD patients had a 1.2 higher risk for developing periodontal disease, which increased to 3.17 for those with prior hospitalization [12]; additionally the presence of periodontal disease is associated with worse pulmonary function in the COPD population [13, 14].

Based on the results of the abovementioned studies one might argue that routine assessment and treatment of periodontal disease could exert a beneficial effect on COPD clinical and functional outcomes. However, while previous systematic reviews have investigated the relationship between periodontitis and COPD, no recent studies have reported on the role of periodontal treatment of COPD outcomes [11, 15–17]. Therefore, the aim of the present study is to systematically review the most recent evidence on the effect of non-surgical or surgical periodontal therapy on quality of life, lung function and exacerbations of patients with COPD and periodontitis.

## Methods

### Protocol and registration

This review was registered in the center for Reviews and Dissemination, University of York, (protocol number: UK #CRD42020158481). The inclusion/exclusion criteria were specified a priori and followed the PICO criteria and Preferred Reporting Items for Systematic Reviews and Meta-Analyses protocol (PRISMA-P) guidelines [18].

### Information sources and search strategy

The following databases were searched by one author (IA) using a combination of keywords for COPD, periodontitis and periodontal therapy: PubMed, Scopus, Virtual Health Library, ScienceDirect, Wiley Online Library, Web of Science, ProQuest Dissertation and Theses Global and Google Scholar (Table 1). Search was limited to papers involving human subjects and published in the English language between 01/01/2000 to 31/03/2020. The original search was created in PubMed and adjusted thereafter to the other databases.

**Table 1:** Search strategy used for each database with the corresponding results

Electronic databases	Search strategy	Limits	Hits
Pubmed	(Periodontitis OR periodontal disease OR gum disease OR periodontal health OR periodontal therapy OR periodontal treatment OR root scaling OR periodontal debridement OR root planing OR oral hygiene) AND (chronic obstructive pulmonary disease OR chronic bronchitis OR emphysema OR COPD)	Publication Date 01/01/2000–31/03/2020 English language Human species	185
Scopus	(Periodontitis OR periodontal disease OR gum disease OR periodontal health OR periodontal therapy OR periodontal treatment OR root scaling OR periodontal debridement OR root planing OR oral hygiene) AND (chronic obstructive pulmonary disease OR chronic bronchitis OR emphysema OR COPD)	Year 2000–2020 English language	55
Virtual Health Library	(Periodontitis OR periodontal disease OR gum disease OR periodontal health OR periodontal therapy OR periodontal treatment OR root scaling OR periodontal debridement OR root planing OR oral hygiene) AND (chronic obstructive pulmonary disease OR chronic bronchitis OR emphysema OR COPD)	Year 2000–2020 English Language	252
ScienceDirect	("Periodontitis" OR "periodontal disease" OR "periodontal therapy" OR "periodontal treatment" OR "root scaling" OR "periodontal debridement" OR "root planing" OR "oral hygiene") AND ("chronic obstructive pulmonary disease")	Year 2000–2020 Article types Research articles	386
Wiley Online Library	(Periodontitis OR periodontal disease OR gum disease OR periodontal health OR periodontal therapy OR periodontal treatment OR root scaling OR periodontal debridement OR root planing OR oral hygiene) AND (chronic obstructive pulmonary disease OR chronic bronchitis OR emphysema OR COPD)	Publication date 01/2000–03/2020 Publication type Journals	213
Web of Science	(Periodontitis OR periodontal disease OR gum disease OR periodontal health OR periodontal therapy OR periodontal treatment OR root scaling OR periodontal debridement OR root planing OR oral hygiene) AND (chronic obstructive pulmonary disease OR chronic bronchitis OR emphysema OR COPD)	Publication Years 2000–2020 Document Types Article Language English	161
Proquest Dissertation and Theses Global	(Periodontitis OR periodontal disease OR gum disease OR periodontal health OR periodontal therapy OR periodontal treatment OR root scaling OR periodontal debridement OR root planing OR oral hygiene) AND (chronic obstructive pulmonary disease OR chronic bronchitis OR emphysema OR COPD)	Publication Date 01/01/2020–31/03/20 Limit to Title and Abstract English Language	124
Google Scholar	("Periodontitis" OR "periodontal disease" OR "periodontal health" OR "periodontal therapy" OR "periodontal treatment" OR "root scaling" OR "root planing" OR "oral hygiene") AND ("COPD" OR "chronic obstructive pulmonary disease")	Limit to Title English language Publication Date 2000–2020	66

### Eligibility criteria

Randomized controlled clinical studies, cross-sectional, cohort and case–control studies reporting data on COPD patients with periodontal disease and its treatment were considered eligible for inclusion. Both prospective and retrospective studies were included and assessed separately. Case reports, case series, and systematic reviews were excluded.

### Definitions, interventions and outcomes measures

COPD diagnosis was based on Global Initiative for Chronic Obstructive Lung Disease report[1], that is a post bronchodilator ratio of forced expiratory volume in 1st second (FEV_1_) to forced vital capacity (FVC) (FEV_1_/FVC < 70%)[1] or on previous track records and physicians’ reports. For the diagnosis of periodontitis, we used the criteria employed by the authors of the individual papers as long as they were validated.

Interventions included non-surgical or surgical periodontal therapy, which was established by hand instruments or ultrasonic devices. The effectiveness of periodontal treatment was assessed according to the results of before-after periodontal treatment studies and from reports which compared COPD patients who received periodontal treatment against those who received no treatment.

The primary outcome measures included the effect of non-surgical, surgical and supportive periodontal therapy on the frequency of COPD exacerbations, lung function (FEV_1_, FEV_1_/FVC) and quality of life. The secondary outcome measures included: oral hygiene and periodontal indices, radiographic alveolar bone loss, levels of inflammatory biomarkers, periodontal bacterial burden, comorbidities, hospitalizations and mortality.

### Data management, study selection and data extraction

Literature search results were uploaded to Distiller Systematic Review (DSR) software, an Internet based software program that facilitates collaboration among reviewers during the study selection process. After duplicates’ removal, the team developed screening questions and forms for level 1 and 2 assessments based on the eligibility criteria. Citation abstracts and screening questions were uploaded to DSR.

Studies were screened by two independent reviewers (IA, AV) who assessed the titles and abstracts of each article. Disagreements were resolved by discussion, but if decision was inconclusive, the assessment of the contentious study continued to full text screening level. At that stage, the same authors carried out the independent appraisal of each article, reasons for exclusion were recorded and in case of disagreement a third author adjudicated unresolved quarrel.

Data were then extracted from each article using a custom data extraction sheet and included the following: principal author, publication year, country, study design, study population, periodontal variables, periodontal therapy techniques, COPD assessment methods and main results.

### Risk of bias in individual studies

The risk of bias of randomized clinical trials was assessed with Cochrane risk of bias tool (RoB2), which assesses the following five domains: bias arising from randomization process, bias due to deviation from intervention, bias due to missing outcome data, bias in measurement of the outcome and bias in selection of reported results. Possible risk of bias judgments were “low risk of bias”, “some concerns” and “high risk of bias” [19].

Risk of bias assessment was performed independently by two review authors (IA and AV) and any disagreement was resolved by discussion. The risk of bias of non-randomized studies was assessed with Cochrane risk of bias tool for non-randomized studies of intervention (ROBINS-I tool) which assesses the following 7 domains of bias: (1) confounding (2) selection of participants into the study (3) classification of interventions (4) deviations from intended interventions (5) missing data (6) measurement of outcomes and (7) selection of the reported result. Possible risk of bias judgments were: “low risk of bias”, “moderate risk of bias”, “serious risk of bias”, “critical risk of bias” and “no information” [20].

### Risk of bias across studies

If a sufficient number of trials were identified (n ≥ 10), analyses were planned to identify reporting biases (“small-study effects” and/or “publication bias”), through the inspection of a contour-enhanced funnel plot and through respective statistical tests for funnel plot asymmetry and identification of missing studies [21].

The overall quality of evidence (confidence in effect estimates) was judged using the Grading of Recommendations Assessment, Development and Evaluation (GRADE) working group methodology [22], according to a recent guidance on combining randomized with nonrandomized studies [23]. A table with a summary of findings was constructed using the format by Carrasco‐Labra et al. [24] GRADEpro software [25] was used to assess the quality of evidence across the domains of risk of bias, consistency, directness, precision and publication bias. Quality was adjudicated as “high” (further research is very unlikely to change our confidence in the effect estimate), “moderate” (further research is likely to have an important impact on our confidence in the effect estimate and may change the estimate), “low” (further research is very likely to have an important impact on our confidence in the effect estimate and is likely to change the estimate) and “very low” (very uncertain about the estimate effect).

## Summary measures and synthesis of results

Data were summarized and considered suitable for pooling if similar groups were reported (or could be formed) and the same outcomes were reported. For dichotomous outcomes risk ratios (RR) with 95% confidence intervals (CIs) would be computed. For continuous outcomes weighted mean differences (95% CIs) or standardized mean differences (95% CIs) would be analyzed. A random-effects model as proposed by DerSimonian and Laird was chosen a priori as the primary method to assess all pooled estimates, as the observed treatment effect was expected to differ across studies due to differences in samples and implementations [26].

The extent and impact of between-study heterogeneity would be assessed by inspecting the forest plots for the localization of heterogeneity, by calculating the I^2^ statistic and by the magnitude and direction of effects. The 95% CIs around I^2^ would be calculated according to the non-central x^2^ approximation of Q. In case of considerable unexplained heterogeneity (I^2^ > 75%) data would be analyzed, but not pooled. If heterogeneity was substantial, a meta-analysis would not be performed, but a systematic narrative synthesis would be provided, presenting data in text and tables to summarize and explain the characteristics and findings of the included studies.

## Results

### Study selection and characteristics

**Fig. 1:**
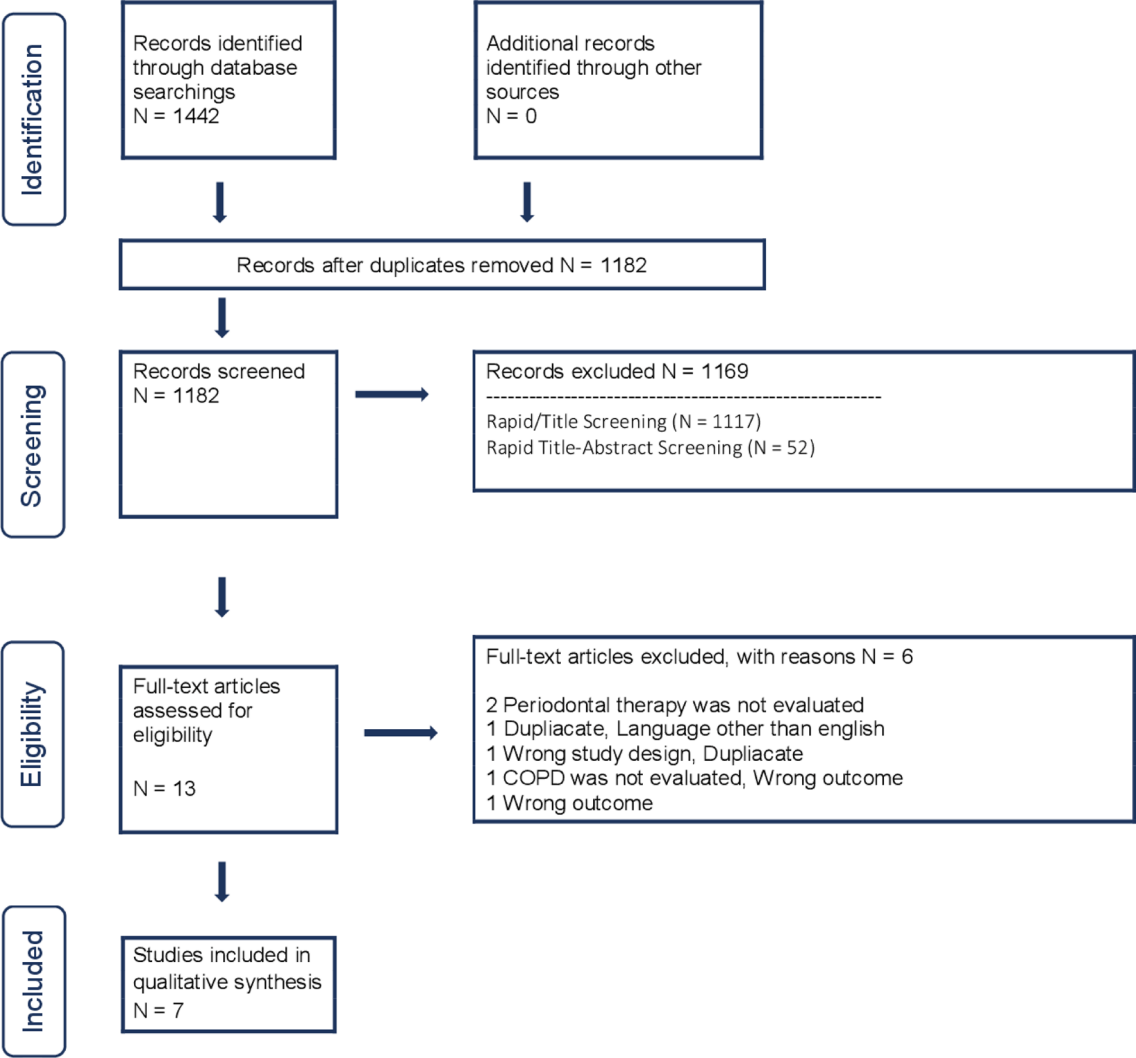
Prisma flow diagram

A flow diagram (Fig. 1) depicts the process that we employed in order to identify relevant articles. Out of 1442 initially retrieved studies, 7 studies were considered eligible for inclusion in this systematic review. Table 2 demonstrates the major characteristics of the 7 studies. Three of the included studies were RCTs [27–29], 2 were case–control studies [30, 31], 1 was cross-sectional study [32] and 1 was a before-after treatment cohort study [33]. These studies were conducted in India [27, 33], Taiwan [30], China [29, 32], Turkey [31] and USA [28] and took place at university and hospital settings. Due to both small sample sizes from most included studies, but most importantly due to differences in the study design, types of interventions and comparisons as well as their methods of reporting results and because of the low quality of the eligible articles, a meta-analysis could not be performed. Hence, the respective results of each study are presented without being statistically processed.

**Table 2:** Characteristics of the studies included in the systematic review. For periodontal outcomes see main text

First author/year/Country [Ref.]	Study design	Study population	Study groups	Periodontal interventions	Outcomes/associations	Follow up period	Results
Das/2017/India [27]	Randomized controlled trial	35 COPD patients with CP	Periodontal treatment, n = 17 No treatment, n = 18	Mouth scaling and root planning with hand instruments versus no treatment	SGRQ	1 year	Treatment group: improvement in the SGRQ “activity” subscore (53.68 ± 16.37 vs. 38.20 ± 13.18; p = 0.005) Control group: no change in SGRQ
Madalli/2016/India [33]	Before-after treatment prospective cohort	30 COPD patients with CP	N/A	Supragingival scaling	FEV_1_/FVC	3–5 months	No significant improvement in FEV_1_/FVC
Shen/2016/Taiwan [30]	Retrospective propensity-matched case–control	11,124 COPD patients with CP	Periodontal treatment, n = 5562 No treatment, n = 5562	Subgingival curettage and root planning and/or invasive periodontal flap surgery versus no treatment	ER visits for COPD exacerbation Hospitalizations for adverse respiratory events ICU admissions All-cause mortality	5 years from inclusion Occurrence of an adverse respiratory event Death Withdrawal from the insurance system	ER visits per 100 person-years for COPD exacerbation: 2.54 ( treatment group) versus 2.88 (control group); adjusted IRR of 0.86 (95% CI 0.78–0.94; p < 0.001) Hospitalizations for adverse respiratory events per 100 person-years: 2.75 (treatment group) versus 3.65 (control group); adjusted IRR 0.74 (95% CI 0.69–0.80; p < 0.001) ICU admissions per 100 person-years: 0.66 (treatment group) versus 0.75 (control group); adjusted IRR 0.84 (95% CI 0.75–0.94; p < 0.01) All-cause mortality per 100 person-years: 1.81 (treatment group) versus 2.87 (control group); adjusted rate ratio 0.57 (95% CI 0.52–0.62; p < 0.001)
Zhou/2014/China [29]	Randomized controlled trial	30 moderate-to-severe COPD patients	Supragingival scaling, root planning and maintenance care (SRP group), n = 20 Supragingival scaling and maintenance care (scaling group), n = 20 No treatment (control group), n = 20	SRP versus scaling versus no treatment	FEV_1_ (% predicted), FEV_1_/FVC Proportion of frequent exacerbations (≥ 2/year)	2 years	*Control group lung function compared to baseline* Lower FEV_1_ at 2 years (56.3 ± 16.4 vs. 51.6 ± 18.4, p < 0.05) Lower FEV_1_/FVC at 1 and 2 years (0.55 ± 0.11 vs. 0.54 ± 0.11 vs. 0.53 ± 0.11; p < 0.05) *SRP versus control group* Higher FEV_1_ at 1 year (55.9 ± 16 vs. 53.6 ± 18.7; p < 0.05) and at 2 years (57.1 ± 19 vs. 51.6 ± 18.4; p < 0.05) Higher FEV_1_/FVC at 1 year (0.59 ± 0.09 vs. 0.54 ± 0.11; p < 0.05) and at 2 years (0.57 ± 0.10 vs. 0.53 ± 0.11; p < 0.05) *Scaling versus control group:* Higher FEV_1_ at 1 year (59.6 ± 17.1 vs. 53.6 ± 18.7, p < 0.005) Higher FEV_1_/FVC at 2 years (0.56 ± 0.11 vs. 0.53 ± 0.11, p < 0.05) *Proportion of frequent exacerbations in SRP versus scaling versus control group* 30% versus 15.8% versus 66.7%; p < 0.004 *Adjusted OR for frequent exacerbations* SRP group: 0.29, (95% CI 0.10–0.84; p = 0.02) Scaling group: 0.004 (95% CI 0.003–0.64; p = 0.02) *No differences in lung function and exacerbations between the 2 treatment groups*
Kucukcoskun/2013/ Turkey [31]	Prospective case–control	40 COPD patients with CP and ≥ 1 exacerbation in the previous year	Periodontal treatment, n = 20 No treatment, n = 20	Full-mouth scaling and root planning with hand instruments and ultrasonic devices	Exacerbation frequency in 12 months Number of hospitalizations	12 months	Exacerbation frequency per patient-year: 1.95 (treatment group) versus 3.25 (control group) Exacerbation frequency decreased in treatment group (3 ± 1.83 vs. 1.95 ± 1.46; p = 0.01) but remained unchanged in the control group (3.5 ± 4.62 vs. 3.25 ± 3.35; p = NS) Hospitalizations increased from 4/year to 7/year in the treatment group and from 10/year to 12/year in the control group
Agado/2012/USA [28]	Randomized controlled trial	30 COPD patients with CP	Periodontal debridement with ultrasonic device, n = 10 Periodontal debridement with hand instruments, n = 10 No treatment, n = 10	Periodontal debridement	SGRQ-A 5-point Likert scale of health status self-perception 7-item illness questionnaire	4–6 weeks	No improvement in SGRQ-A, health status self-perception and illness questionnaire post treatment
Liu/2012/China [32]	Cross-sectional	392 COPD patients	Frequent exacerbators (≥ 2 events/year), n = 183 Infrequent exacerbators (< 2 events/year), n = 209	Supragingival scaling ≥ 1/year, n = 15 < 1/year, n = 377	Correlation between periodontal/oral health and its treatment and frequency of COPD exacerbations	N/A	*Risk factors for frequent exacerbations* ≤ 25 remaining teeth (adjusted OR 1.69, 95% CI 1.03–2.77; p = 0.04) < 1 daily brushing frequency (adjusted OR 4.19, 95% CI 1.44–12.1; p = 0.008)

*COPD* chronic obstructive pulmonary disease, *CP* chronic periodontitis, *ER* emergency room, *GOLD* global initiative for chronic obstructive lung disease, *ICU* intensive care unit, *IRR* incidence rate ratio, *N/A* not applicable, *OR* odds ratio, *SGRQ* St. George Respiratory Questionnaire, *SGRQ-A* American English modified SGRQ, *SRP* scaling and root planning

The diagnosis of COPD was based on GOLD criteria [27, 29, 31–33], medical records [28] and ICD-9 classification [30]. The diagnosis of COPD exacerbations was based on the GOLD criteria [29, 32], ICD-9 classification [30] and symptom worsening requiring treatment adjustment and/or hospitalization [31]. COPD exacerbations were reported by the patients [32], by the investigators [29] or were reported by the patient and confirmed by the investigator [31]; while 2 studies have also provided data on COPD exacerbations prior to periodontal interventions based on patients’ recall [29, 31].

The diagnosis of periodontitis was based on a mean attachment loss (MAL) ≥ 1.5 mm[28], a community periodontal index (CPI) > 3 and a loss of attachment (LOA) > 1[33], the presence of at least one site with a pocket probing depth (PPD) > 3 mm and periodontal clinical attachment loss (CAL) > 3 mm[29], the ICD-9 classification[30], the American Academy of Periodontology Workshop 1999 guidelines[31, 34] and on oral/periodontal health indices [27, 32]. Quality of life was assessed with the Saint George Respiratory Questionnaire (SGRQ) in 2 studies [27, 28]. The effect of periodontal treatment on lung function variables was investigated in 2 other studies [29, 33].

Periodontal therapy was established through scaling and root planning using hand instruments [27], supragingival scaling, hygiene instructions and preprocedural antibiotics [33], subgingival curettage, root planning and periodontal flap surgery (if needed) [30], oral hygiene instructions, supra- and subgingival scaling using hand instruments and ultrasonic devices [28, 29, 31]. In another study, information about the frequency of previous supragingival scaling procedures was obtained by interview [32].

### Risk of bias within studies

**Fig. 2:**
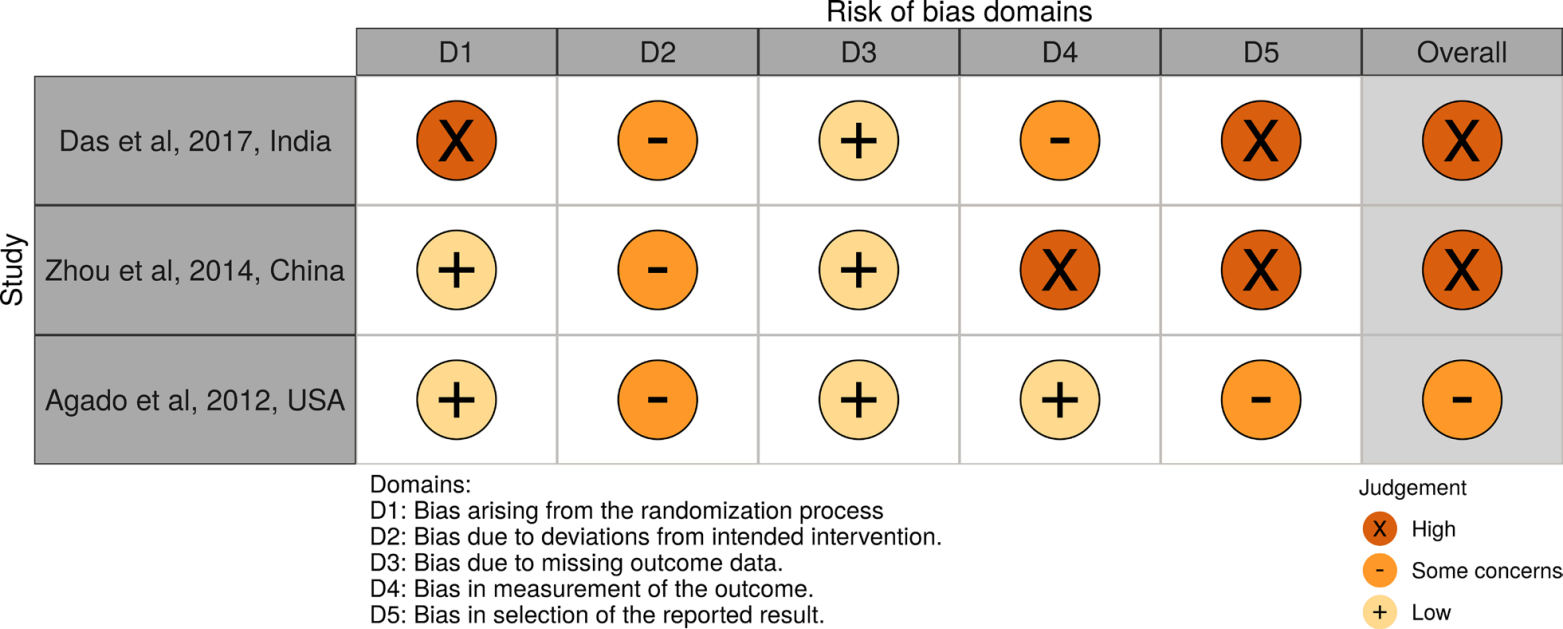
Risk of bias assessment for randomized trials (RoB2)

**Fig. 3:**
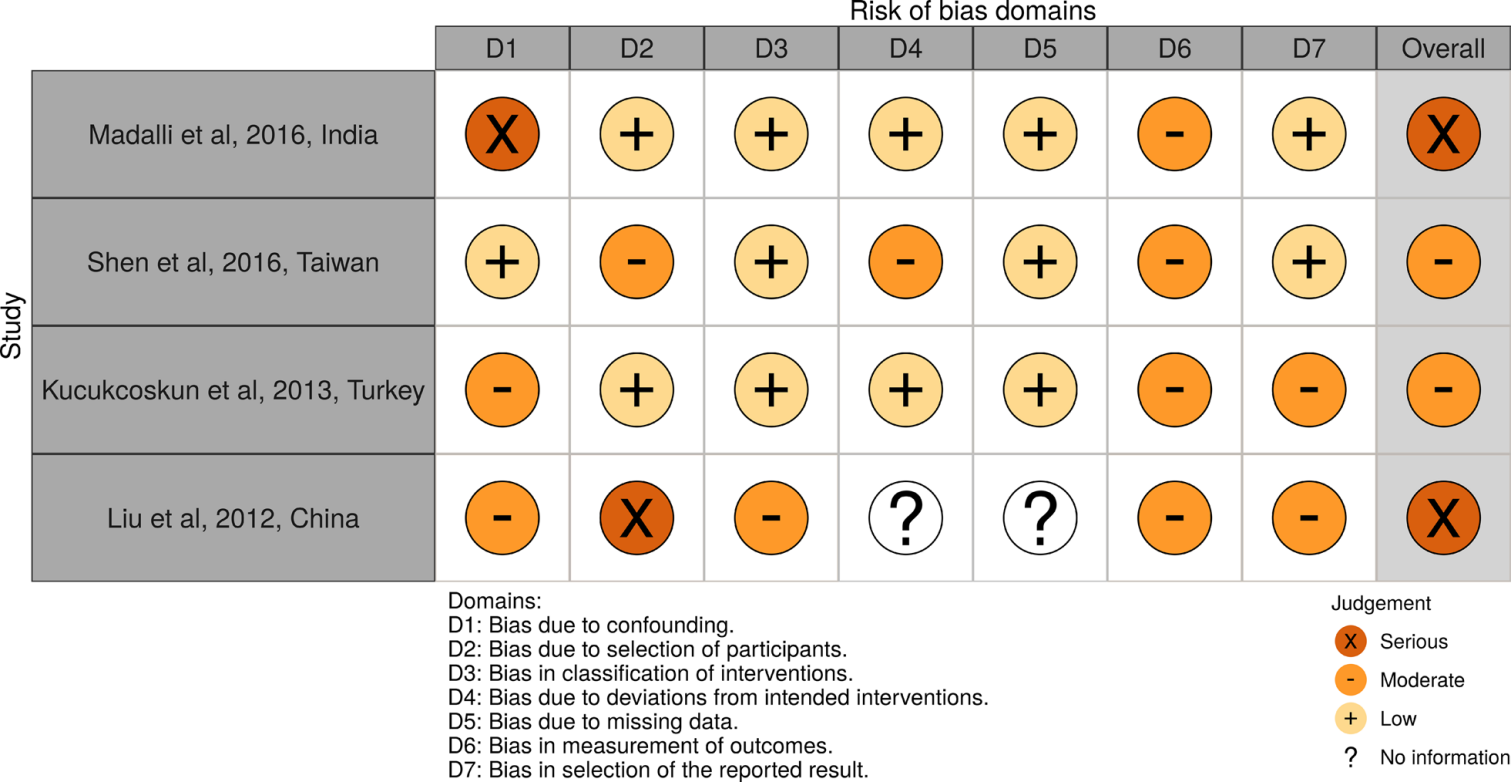
Risk of bias assessment for non-randomized studies (ROBINS-I)

Risk of bias for RCTs was judged as “some concerns” in one trial[28] and “high” in two trials [27, 29]. With regard to the rest of the studies, serious methodological limitations were found for two studies [32, 33] and moderate for another two [30, 31]. Risk of bias summary plots are depicted in Figs. 2 and 3.

## Results of data synthesis

### Quality of life

Two studies [27, 28] evaluated the effect of periodontal therapy on quality of life. Das et al. [28] randomized 35 COPD patients (diagnosed according to the GOLD spirometric criteria) to receive either periodontal treatment (treatment group, n = 18) or no treatment (control group, n = 17). Interventions included full-mouth scaling and root planning with hand instruments as well as oral hygiene instructions, while the effect of periodontal therapy on quality of life was assessed with the SGRQ score. All patients were reassessed at 1-year post-treatment for significant changes in periodontal and oral parameters and in SGRQ scores. With respect to the intervention group in this study significant improvements were observed in the Oral Hygiene Index-Simplified (OHI-S), Gingival Index (GI) and CAL, but not in PPD, while no differences in any of the appraised parameters were noted in the control group. The SGRQ “activity” subscore significantly improved in the treatment group post treatment (53.68 ± 16.37 vs. 38.20 ± 13.18; p = 0.005), but no differences in any of the SGRQ subscores were observed in the control group. At the end of the 1-year follow-up period, no differences were noted in the SGRQ subscores between the two groups with the exception of the “activity” subscore which was significantly better in the intervention group (38.20 ± 13.18 vs. 56.48 ± 18.31; p < 0.01).

Agado et al. [28] randomized 30 COPD patients (recruited from medical databases) with chronic periodontitis (MAL ≥ 1.5 mm) into periodontal therapy with ultrasonic instrumentation (n = 10), periodontal therapy with hand instruments (n = 10) or no treatment (n = 10) for a follow-up period of 4–6 weeks; periodontal treatment was completed when all clinically detectable deposits had been removed. The effect of periodontal therapy on quality of life was evaluated with the American English modified SGRQ score (SGRQ-A), while patients graded their perception of health status on a 5-point Likert scale from “very poor” to “very good”. In addition, all participants provided a yes/no response to a customary made 7-item “illness questionnaire” which interrogated respiratory or other illness within the previous 4 weeks, physician visits, antibiotic use, use of respiratory medications, respiratory problems after dental care and avoidance of dental care due to respiratory ailments. All questionnaires were issued by an investigator blinded to the type of intervention. At the end of the follow-up period no significant difference was noted between the 3 groups in SGRQ-A total scores, self-assessment of health scores and “illness questionnaire” and no improvement between pre- and post-test. However, each group showed a significant improvement in the SGRQ-A “activity” subscore but with no interactions between groups.

### Lung function

Two studies [29, 33] evaluated the effect of periodontal therapy on lung function. Madalli et al.^29^ conducted a before-after treatment prospective cohort study of 30 COPD patients with periodontal disease to investigate the effect of periodontal treatment on FEV_1_/FVC variables. COPD diagnosis was based on the GOLD spirometric criteria and the diagnostic criteria for periodontal disease included a CPI score ≥ 3 and a LOA score ≥ 1. Interventions included supragingival scaling and oral hygiene instructions and all patients were re-assessed after 3–5 months. This study observed no significant changes in CPI and LOA scores, while no significant improvement in FEV_1_/FVC was also noted (48.08 ± 12.01 vs. 51.25 ± 12.29; p = NS). Zhou et al. [29] randomized 60 moderate-to-severe COPD patients (diagnosed according to the GOLD criteria) into scaling, root planning and maintenance care (SRP group, n = 20), supragingival scaling alone and maintenance supragingival scaling (scaling group, n = 20) or no treatment (control group, n = 20). Periodontal disease was considered based on the presence of at least one site with a PPD > 3 mm and a CAL > 3 mm. All patients were re-assessed for changes in periodontal variables, namely PPD, CAL, bleeding index (BI) and plaque index (PI) at 6 months, 1 year and 2 years, while lung function variables (FEV_1_, FEV_1_/FVC) were re-evaluated at 1 year and 2 years. This trial observed significant improvements in both treatment groups at 6 months, 1 year and 2 years for all the appraised periodontal parameters, whereas no improvements were noted in the control group; all periodontal variables were worse in the control group as compared to each of the treatment groups at any of the investigated time points. With respect to the lung function variables, when compared to baseline, the control group exhibited a significant decrease in FEV_1_ (% predicted) at 2 years (56.3 ± 16.4 vs. 51.6 ± 18.4, p < 0.05) and in FEV_1_/FVC at 1 and 2 years (0.55 ± 0.11 vs. 0.54 ± 0.11 vs. 0.53 ± 0.11, respectively; p < 0.05). When compared to the control group, the SRP group showed significantly improved levels of FEV_1_ (% predicted) and FEV_1_/FVC at 1 year (55.9 ± 16 vs. 53.6 ± 18.7 and 0.59 ± 0.09 vs. 0.54 ± 0.11, respectively; p < 0.05) and at 2 years (57.1 ± 19 vs. 51.6 ± 18.4 and 0.57 ± 0.10 vs. 0.53 ± 0.11, respectively; p < 0.05). Likewise, when the scaling group was compared to controls, it exhibited a significantly higher FEV_1_ (% predicted) at 1 year (59.6 ± 17.1 vs. 53.6 ± 18.7, p < 0.005) and FEV_1_/FVC at 2 years (0.56 ± 0.11 vs. 0.53 ± 0.11, p < 0.05). No differences in lung function were observed between the two treatment groups at any of the abovementioned time points.

### COPD exacerbations

Four studies [29–32] evaluated the effect of periodontal therapy on the frequency of COPD exacerbations. In the previously mentioned RCT by Zhou et al., COPD exacerbations post-randomization were defined according to the GOLD criteria and their frequencies were assessed at 6 months, 1 year and 2 years, while their frequencies during the last year prior to randomization were self-reported by the patients; patients with ≥ 2 events /year were defined as “frequent” exacerbators. At 2 years, the SPR and the scaling groups showed a lower proportion of “frequent” exacerbations as compared to controls (30% vs. 15.8% vs. 66.7%, respectively; p < 0.004). In a multivariate logistic regression model, the calculated OR for “frequent” exacerbations was 0.29 (95% CI 0.10–0.84; p = 0.02) for the SRP group and 0.004 (95% CI 0.003–0.64; p = 0.02) for the scaling group after adjusting for age, gender, body mass index (BMI) and baseline frequency of exacerbations. No differences were noted in the frequency of COPD exacerbations between the two treatment groups.

Shen et al. [30] conducted a population-based, retrospective case–control study based on the Taiwan National Health Insurance claims data. COPD and periodontal disease diagnosis, periodontal interventions and adverse respiratory effects were identified based on the database’s ICD-9 classification. The study population consisted of 5562 COPD individuals that received periodontal therapy and 5562 propensity-matched controls. All subjects were followed up to a maximum of 5 years or until death, occurrence of an adverse respiratory event or withdrawal from the insurance system. Periodontal interventions included supragingival scaling and root planning as well as periodontal flap surgery, depending on the severity of the disease. The primary outcomes included emergency room (ER) visit and hospitalization rates following acute COPD exacerbation, pneumonia or acute respiratory failure. Overall, all types of adverse respiratory events were less frequent in the treatment group as opposed to the control group. After adjusting for confounders, including age, gender, residential area, occupation, income and comorbidities, the ER visit rate per 100 person-years for COPD exacerbation was 2.54 for the treatment group versus 2.88 for the control group, for an adjusted incidence rate ratio (IRR) of 0.86 (95% CI 0.78–0.94; p < 0.001).

Kucukcoskun et al. [31] conducted a prospective case–control study consisting of 40 patients with COPD, diagnosed according to the GOLD criteria with a history of ≥ 1 exacerbation during the previous year and with moderate-to-severe chronic periodontitis based on the 1999 American Academy of Periodontology Workshop guidelines. COPD exacerbation was defined as a worsening in the baseline respiratory symptoms lasting at least 2 days and necessitating oral steroids and antibiotics (moderate exacerbation) or hospitalization (severe exacerbation). Patients were allotted to periodontal treatment (oral hygiene, full mouth scaling and root planning with hand instruments and ultrasonic devices completed over 3 visits, n = 20) or no treatment (n = 20) according to their access to periodontal services. Exacerbations were self-reported by the patients and confirmed by the investigator, while the number of exacerbations and hospitalizations during the last year before enrollment was also registered. Periodontal procedures were performed by a dentist blind to the study design and COPD severity and all patients were re-evaluated at 6 and 12 months. With respect to the periodontal parameters, when compared to baseline, the treatment group exhibited significant improvements in BP, GI, PPD and CAL at 6 months; at 12 months, this improvement remained significant only for CAL. On the other hand, in the control group most of the periodontal parameters showed significant deterioration at 6 (GI, PPD, PI and CAL) and 12 months (GI, PPD, and CAL). The frequency of exacerbations per patient-year was 1.95 events in the treatment group versus 3.25 events in the control group (p = 0.01). After 1 year of follow-up, exacerbation frequency, was significantly reduced in the treatment group (3 ± 1.83 vs. 1.95 ± 1.46; p = 0.01) but remained unchanged in the control group (3.5 ± 4.62 vs. 3.25 ± 3.35; p = NS).

Liu et al. [32] conducted a cross-sectional study investigating the role of periodontal health on the frequency of COPD exacerbations. The diagnosis and definition of COPD exacerbation were based on GOLD criteria. Overall, 392 COPD patient were assessed for the presence and severity of periodontal disease and were interviewed about oral health habits, history of dental visits and frequency of supragingival scaling procedures (≥ 1 time/year vs. < 1 time/year) during the last 12 months. Periodontal examination was conducted by an investigator blinded to COPD severity. Patients also provided information about the frequency of exacerbations during the previous 12 months with patients with < 2 events classified as “infrequent” exacerbators (n = 209), whereas those with ≥ 2 events considered “frequent” exacerbators (n = 183). In a multivariate logistic regression model adjusted for age, gender, BMI and smoking, ≤ 25 remaining teeth (adjusted OR 1.63, 95% CI 1.01–2.61; p = 0.045), a PI > 2 (adjusted OR 1.93, 95% CI 1.09–3.42; p = 0.02) and < 1/day brushing frequency (adjusted OR 4.5, 95% CI 1.6–12.7; p = 0.004) were the independent predictors of “frequent” exacerbations. When COPD severity and dyspnea scores were additionally considered in the model, this association was still significant only for fewer remaining teeth (adjusted OR 1.69, 95% CI 1.03–2.77; p = 0.04) and lower daily brushing frequency (adjusted OR 4.19, 95% CI 1.44–12.1; p = 0.008). The frequency of supragingival treatment was not included in any of the models, however the number of patients who underwent frequent treatment (≥ 1 time/year) was very low (4%).

### Secondary outcomes

#### Periodontal outcomes

Regarding the periodontal outcomes, the appraised parameters presented amelioration or no change. In the RCT by Das et al., periodontal therapy with full-mouth scaling and root planning with hand instruments combined with oral hygiene was associated with improvements in OHI-S (3.38 ± 1.27 vs. 2.32 ± 1.21; p = 0.018), GI (1.79 ± 0.54 vs. 1.19 ± 0.47; p = 0.001) and CAL (4.17 ± 0.75 vs. 3.26 ± 0.90; p = 0.003) at 1 year post-treatment [27]. In the case–control study by Kucukcoskun et al. full mouth scaling and root planning with hand instruments plus oral hygiene effected improvements in BOP% (58 ± 28 vs. 47 ± 19; p < 0.01), GI (1.52 ± 0.37 vs. 1.34 ± 0.23; p < 0.01), PPD (2.70 ± 0.66 vs. 2.39 ± 0.41; p < 0.01) and CAL (3.73 ± 1.16 vs. 3.39 ± 1.01; p < 0.01) at 6 months, as compared to baseline; at 12 months CAL continued to show significant improvement with respect to its baseline value (3.73 ± 1.16 vs. 3.52 ± 1.02; p < 0.01) [31]. Likewise, in the RCT by Zhou et al. [29], all assessed periodontal parameters (i.e. PD, CAL, BI, PI), exhibited significant improvements for both the SRP and the scaling group at any time point (6 months, 1 year, 2 years). On the contrary, supragingival scaling plus oral hygiene did not result in any improvement in CPI and LOA scores (4 ± 0.51 vs. 4.0 ± 0.48 and 2 ± 0.37 vs. 2 ± 0.37, respectively; p = NS) after 3–5 months as reported by Madalli and colleagues. Radiographic alveolar bone loss and levels of inflammatory biomarkers were not assessed by any of the included studies.

#### Periodontal pathogens, hospitalizations, mortality and comorbidities.

In the study by Madalli et al. [33] the proportion of COPD patients with a positive sputum sample for *Porphyromonas gingivalis* decreased from 14/30 to 8/30 at 3–5 months post treatment, although this finding was not significant. In the case–control study by Kucukcoskun et al. [31], 1-year hospitalization rate post-treatment increased as compared to the previous year both in the treatment (from 4 to 7 events) and in the control group (from 10 to 12 events). On the other hand, in the retrospective case–control study by Shen et al. [30] the respective hospitalization rates per 100 person-years for all types of adverse respiratory events were 2.75 for the treatment group versus 3.65 per 100 person-years for the control group (adjusted IRR 0.74, 95% CI 0.69–0.80; p < 0.001). In addition, ICU admissions per 100 person-years were 0.66 for the treatment group versus 0.75 for the control group (adjusted IRR 0.84, 95% CI 0.75–0.94; p < 0.01), while the all-cause mortality rate was 37% lower for those treated for periodontal disease (adjusted rate ratio 0.57, 95% CI 0.52–0.62; p < 0.001). No study reported on the effects of periodontal treatment on the prevalence and severity of COPD-associated comorbidities.

### Risk of bias across studies

The quality of evidence for all outcomes ranged from moderate to very low (Table 3). The main reason for downgrading the quality of evidence pertained to the inclusion of non-randomized studies with serious methodological issues that most probably introduce bias and to the imprecision of estimates due to narrative synthesis. This means that further research with well-designed studies is very likely to have an important impact, and is likely to change our current estimates of effect.

**Table 3:** Summary of findings table according to GRADE approach

Periodontal therapy compared to no periodontal therapy in patients with PD and COPD			
Patient or population: patients with PD and COPD Setting: university clinics and Hospitals (India, Taiwan, China, Turkey and USA) Intervention: periodontal therapy Comparison: no periodontal therapy			
Outcome no. of participants (studies)	Impact	Certainty	What happens with periodontal therapy
Exacerbations of COPD assessed with: exacerbation frequency No. of participants: 11,616 (4 studies)	In one study, acute exacerbation rate was 2.54 versus 2.88 per 100 person-years for emergency room use, [adjusted IRR = 0.86 (95% CI 0.78–0.94, p < 0,001)] and 1.36 versus 1.71 per 100 person-years for hospitalizatios, [adjusted IRR = 0.78 (95% CI 0.72–0.85, p < 0,001)] In another study, proportion of frequent exacerbations was statistical significantly (p < 0.004) lower in periodontal therapy group at 2-years follow up In the third study, frequency of exacerbations was 1.95 per patient-years compared to 3.25 in the control group (p = 0.01) In the last study, although supragingival scaling < 1time/year was associated with higher proportion of patients with frequent exacerbations, in the adjusted model no statistical significance was obtained (OR = 2.23, 95% CI: 0.58–8.59, p = 0.24)	⨁⨁⨁◯ MODERATE^a,b,c^	Probably reduces frequency of exacerbations of COPD
Quality of life assessed with: SGRQ follow up: range 4 weeks to 1 years No. of participants: 65 (2 studies)	In one study SGRQ domain scores were lower in study group at one year follow up(p < 0.05 only for activity domain score) In another study, SGRQ and illness Questionnaire responses showed no significant difference between groups	⨁◯◯◯ VERY LOW^c,d^	Too heterogenous response to synthesize across studies
Lung function assessed with: FEV1, FEV1/FVC follow up: range 3 months to 2 years No. of participants: 116 (2 studies)	In one study, although FEV1/FVC mean value increased, no statistical significance was obtained In another study, FEV1 increased statistical significantly in one year follow up for both periodontal therapy groups(p = 0.03), but only for SRP group in two years follow up. FEV1/FVC increased statistical significantly in two years follow up for both periodontal therapy groups(p = 0.02), but only for SRP group in one year follow up (p = 0.04)	⨁◯◯◯ VERY LOW^c,e^	Too heterogenous response to synthesize across studies

^a^Downgraded by one level for bias due to the inclusion of non-randomized study with high risk of bias

^b^Inconsistency attributed to the fact that exacerbation frequency was expressed by different values(exacerbations per 100persons-years, exacerbations per patient-year, proportion of exacerbation, proportion of patients with exacerbations) was not serious to downgrade the quality of evidence for this outcome

^c^Narrative synthesis was conducted. Estimates are not precise

^d^Downgraded by two levels for bias, because one of the two included studies is non-randomized with high risk of bias and serious limitations

^e^Included studies are judged as "high" and "serious" risk of bias

## Discussion

### Summary of evidence

The present systematic review addresses the effects of non-surgical or surgical periodontal treatment on various clinical outcomes of patients suffering from COPD and periodontal disease. To the best of our knowledge this is the first study to systematically assess the direct effect of periodontal therapy on the outcomes of patients with COPD. In summary, the accumulated evidence suggests that effective periodontal treatment in patients with COPD and periodontitis is associated with a reduced frequency of COPD exacerbations and slower lung function decline, although it is unclear whether it can also improve the quality of life. In addition, based on the results of a large population-based retrospective case–control study, periodontal treatment reduces the rate of hospitalizations for respiratory events (including COPD exacerbations) and is associated with lower all-cause mortality.

COPD is associated with systemic inflammation [35] and oxidative stress [36] and is now considered a systemic disease with frequent manifestations “outside the lungs” [37]. Thus, several comorbidities including cardiometabolic ones, lung cancer, osteoporosis, anxiety and depression, obstructive sleep apnea and gastroesophageal reflux occur more frequently in COPD patients compared to the general population [38]. Considering these findings, there is an ongoing need to further identify relevant comorbidities which share common pathogenetic factors with COPD and thus could exert a negative effect on its clinical outcomes. A growing body of studies over the last years has established an association between periodontitis and COPD [11, 15–17]. Both diseases are characterized by neutrophilic inflammation with subsequent proteolytic destruction of connective tissue and share common risk factors such as age, smoking and social deprivation [9]. Accumulating evidence has identified several putative pathophysiologic mechanisms linking the two disorders including aspiration of cytokines, bacteria and neutrophils [9, 10], hematogenous dissemination of periodontal pathogens via gingival micro-ulcerations [39] and hyper-reactivity of peripheral blood neutrophils to bacterial stimuli and reactive oxygen species [40]. Among these, the most compelling evidence supports the hypothesis of neutrophilic inflammation and neutrophil-mediated tissue damage [9, 10]. Although a direct causal link cannot be unequivocally suggested based on current evidence, some studies have shown a higher loss of lung function in patients with more severe periodontal disease^34^. Interestingly, COPD patients receiving corticosteroids (CS) are exposed at higher risk for periodontal disease as compared with those who are not; an additional issue to consider regarding the puzzling associations between COPD and periodontitis and specifically the additive effects of COPD treatment on onset and progression of periodontal disease [41, 42].

Several mechanisms have been proposed regarding the beneficial effects of periodontal treatment on COPD outcomes. First, periodontal therapy has been shown to reduce the concentration of various inflammatory biomarkers, potentially leading to less severe inflammatory response in patients with COPD [43, 44]. Second, removal of dental plaque may lead to a reduction of oral mucosa colonization with pathogens which are commonly isolated in respiratory and dental specimens of patients with COPD, especially during exacerbations [45, 46]. On the other hand, some authors have reported a deterioration of respiratory symptoms due to bacterial contamination caused by aerosols produced during dental treatment with ultrasonic devices [33, 47]. Antimicrobial preprocedural mouth rinse, high volume evacuation suction and air polishing avoidance have been proposed as precautionary measures [47].

### Clinical relevance

The present systematic review underscores the need for collaboration between pulmonologists and dental practitioners in the field of COPD management. Poor oral health is one of the primary risk factors for COPD exacerbations and could stand on its own right as a potentially modifiable condition [48, 49]. On the other hand, the presence of periodontal disease should alert dental practitioners to the possibility of coexistent COPD and prompt them to refer the patient for further evaluation [29]. Future clinical trials should aim to investigate whether COPD patients will benefit from routine oral/dental assessment, and to provide important insights on the pathophysiological link between periodontal disease and COPD.

### Strengths and limitations

This systematic review was conducted according to an a priori registered protocol and has several strengths, including a comprehensive literature search, inclusion of randomized and non-randomized studies and the application of the GRADE approach to assess the overall quality of the evidence.

However, there are also several limitations that should be acknowledged. First, our search strategy was restricted to the last 20 years. However, it was our aim to evaluate the most recent data pertaining to the impact of periodontal treatment on COPD outcomes given that the most widely accepted diagnostic criteria for periodontitis and COPD were proposed in 1999 [34] and in 2001 [50], respectively and continue to be in use. In addition, the overall quality of evidence was very low for the effect of periodontal therapy on lung function and quality of life and moderate regarding its effect on COPD exacerbation frequency. Other limitations include the differences in study design and their methods of reporting results, the lack of uniformly applied definitions for COPD and periodontitis and the moderate-to-high risk of bias in individual studies. It should also be noted that in some studies exacerbations were self-reported by patients and not objectively verified by the investigator. Importantly, neither of the studies made adjustments for COPD severity according to FEV_1_, nor explored the effect of periodontal treatment on respiratory symptoms, both considered powerful predictors of future exacerbations and hospitalizations [51]. Of note, exacerbations and their related risk of hospitalization are strongly associated with greater lung function decline among COPD patients [52]. Interestingly, the study by Shen et al. [30] demonstrated a beneficial role of periodontal treatment both on exacerbation rate and on FEV_1_ decline_,_ a finding that should be examined more concisely in future studies. Additionally, only one study investigated the role of supportive follow-up periodontal therapy on COPD outcomes [29, 53]. Finally, the impact of periodontal treatment on prevalence and severity of COPD comorbidities has not been investigated.

## Conclusions

Very low-to-moderate quality evidence suggests that periodontal treatment is associated with slower lung function decline, reduced frequency of exacerbations and less use of healthcare resources in patients with COPD and chronic periodontitis. Based on these findings we recommend that all COPD patients should be advised to undergo regular dental examination and follow up. However, because of the limitations of the available evidence, large-scale clinical trials are warranted to further elucidate the effect of periodontal treatment on COPD outcomes.

## Supplementary Information

**Additional file 1: TableS1**. Excluded studies with reasons.

## Data Availability

The datasets used and/or analyzed during the current study available from the corresponding author on reasonable request.

## Abbreviations

COPD: Chronic obstructive pulmonary disease
RoB2: Risk of bias 2 tool
ROBINS-I: Risk of bias for non-randomized studies of intervention
GRADE: Grading of Recommendations Assessment, Development and Evaluation
PROSPERO: International prospective register of systematic reviews
CVD: Cardiovascular disease
PICO: Participants, Interventions, Comparisons, Outcomes
PRISMA-P: Preferred Reporting Items for Systematic Reviews and Meta-Analyses protocol
FEV_1_: Forced expiratory volume in 1st second
FVC: Forced vital capacity
DSR: Distiller systematic review software
RR: Risk ratio
CI: Confidence interval
RCT: Randomized controlled trial
MAL: Mean attachment loss
CPI: Community periodontal index
LOA: Loss of attachment
PPD: Periodontal pocket depth
CAL: Clinical attachment loss
ICD-9: International classification of diseases 9th clinical modification
OHI-S: Oral hygiene index simplified
GI: Gingival index
BI: Bleeding index
PI: Plaque index
BMI: Body mass index
CP: Chronic periodontitis
ER: Emergency room
GOLD: Global initiative for chronic obstructive lung disease
ICU: Intensive care unit
IRR: Incidence rate ratio
N/A: Not applicable
OR: Odds ratio
SGRQ: St. George Respiratory Questionnaire
SGRQ-A: American English modified SGRQ
SRP: Scaling and root planning
