# Supplementary material for: Effect of periodontal therapy on COPD outcomes: a systematic review
Source: BMC Pulm Med. 2021 Mar 18;21:92. doi: 10.1186/s12890-021-01429-2 (PMC7976708; doi:10.1186/s12890-021-01429-2)
Supplement: Supplementary file 1 — Additional file 1: TableS1. Excluded studies with reasons. [file 12890_2021_1429_MOESM1_ESM.docx]

**Table S1: Excluded studies with reasons**

| Article | Exclusion reason |
| --- | --- |
| Zhou X et al, 2019. Influence of non-surgical periodontal treatment on the quality of life in chronic obstructive pulmonary disease patients with chronic periodontitis. | Duplicate  Language other than English |
| Hesham A et al, 2018. Chronic obstructive pulmonary disease exacerbations and periodontitis: a possible association. | Periodontal therapy was not evaluated |
| Bellissimo-Rodrigues WT et al, 2018. Is it necessary to have a dentist within an intensive care unit team? Report of a randomized clinical trial. | Wrong outcome |
| Santos S.R. et al, 2017. Effects of periodontal treatment on exacerbation frequency and lung function in patients with chronic periodontitis: Study protocol of a 1-year randomized controlled trial. | COPD was not evaluated  Wrong outcome |
| Agado B. and Bowen D., 2010. Health-Related Quality of Life and illness following periodontal instrumentation for patients with chronic obstructive pulmonary disease and chronic periodontitis | Wrong study design  Duplicate |
| Al-Jawder, Mareya, 2003. Oral health of patients suffering from chronic obstructive pulmonary disease and its relationship with the exacerbation events. | Periodontal therapy was not evaluated |
